# Fracture Resistance of Sintered Monolithic Zirconia Dioxide in Different Thermal Units

**DOI:** 10.3390/ma15072478

**Published:** 2022-03-27

**Authors:** Andrea Ordoñez Balladares, Cristian Abad-Coronel, Joao Carlos Ramos, Benjamín José Martín Biedma

**Affiliations:** 1 Faculty of Dentistry, Universidad de Guayaquil, Guayaquil 090514, Ecuador; andrea.ordonezb@ug.edu.ec; 2 Faculty of Dentistry, Universidad de Santiago de Compostela, 15782 Galicia, Spain; benjamin.martin@usc.es; 3 Department of Digital Dentistry and CAD/CAM Materials, Faculty of Dentistry, Universidad de Cuenca, Cuenca 010107, Ecuador; 4 Faculty of Dentistry, Universidad San Francisco de Quito, Quito 170901, Ecuador; 5 Faculty of Medicine, Universidad de Coimbra, 3000-370 Coimbra, Portugal; joao.ramos@ipmd.pt

**Keywords:** zirconia, sintering, CAD/CAM materials, fracture strength

## Abstract

The purpose of this study was to compare the fracture strength of monolithic zirconium dioxide subjected to a sintering process in two different furnaces: InFire HTC Speed and CEREC SpeedFire. Methods: Forty restorations were designed and machined using a computer aided design / computer aidded machine (CAD/CAM) system. The restorations were randomly assigned to two groups of 20 samples each, Group 1 for the SpeedFire furnace (fast sintering) and Group 2 for the InFire furnace (slow sintering). Each of the crowns was subjected to a maximum compression load recorded in Newtons (N) and a displacement control with a speed of 1 mm/min. Results: Group 1 presented an average of 1222.8 N and a standard deviation of 136.91 N. Group 2 obtained a mean of 1068.5 N and a standard deviation of 316.39 N. Conclusions: There were no significant differences between the two groups, and the mechanical strength of the material was not affected, which would imply a saving of clinical and laboratory time when performing rapid sintering on monolithic translucent zirconium dioxide restorations. However, rapidly sintered restorations have limited reliability.

## 1. Introduction

Currently, high-strength ceramic materials and systems have been developed thanks to the introduction of CAD/CAM technologies, with wide use in restorative treatments, in both the anterior and posterior sectors. In addition, there is a trend in dental clinics to perform minimally invasive procedures for rapid fabrication of dental restorations, with adequate biomechanical performance [1,2]. One of the most commonly used materials at present is zirconium dioxide, with which restorations can be fabricated from pre-sintered blocks with sizes up to 25% larger and which after milling are sintered at a temperature of around 1450 °C [3,4]; after this process, they are reduced to the size foreseen in the design.

Zirconium is an unstable and polymorphic material that, depending on its temperature, can produce three different crystallographic patterns or phases: Monoclinic when it reaches a temperature up to 1170 °C, being quite thermodynamically stable; tetragonal when it reaches up to 2370 °C; and cubic above 2370 °C up to the melting point. When zirconium undergoes cooling, there is a spontaneous transformation from the tetragonal to the monoclinic phase, with an increase in crystal volume (4–5%), sufficient to cause catastrophic failure [5]. At the beginning of the last century, it was discovered that zirconium can remain stable at room temperature after adding cubic oxides called stabilizers [5,6]. The volumetric expansion is restricted by the presence of the surrounding materials, resulting in a compressive force acting on the crack and hindering its propagation. The tetragonal structure allows the fracture toughness of zirconia ceramics and the control of induced stress [7]. Several generations of zirconia have emerged from the incorporation of certain materials into its composition to improve its optical characteristics, decreasing as little as possible its mechanical characteristics (Table 1). There are varieties of zirconia that change according to the amounts of additives or dopants added [8]. The most commonly used is the one partially stabilized with yttrium oxide (3 mol %), which has low porosity and high density [9].

Zirconium dioxide-based restorations provide a balance of versatility and simplicity. Initially, because of the opaque nature of zirconia, zirconia copings were layered with feldspathic ceramics to achieve a better aesthetic appearance, which had a negative influence on the longevity of this type of restoration as they tended to chip in the veneer layer [11]. As mentioned by Kanat et al. [12], the bond strength between the ceramic veneer and the zirconia framework is the weakest component of layered frameworks. With the consolidation of CAD/CAM techniques and digital flows, monolithic zirconia blocks with excellent mechanical properties emerged to allow restorations with lower chipping rates [13].

Besides containing zirconium oxide (≥99.0%) in their chemical composition, zirconia blocks have yttrium oxide (4.5–6%), hafnium dioxide (≤5%), aluminum oxide (≤0.5%), and other oxides (≤0.5%). This material has a density of 6.08 ± 0.2 g cm^−3^, a flexural strength greater than 900 MPa, and a fracture toughness of ≥6 to 10 MPa.m^1/2^. As for the color, this type of translucent monolithic zirconium dioxide material is not pre-colored and corresponds to a bleached shade, so it is possible to place dyes or stains on restorations made of this material [5,9,13,14].

Crystalline materials can exhibit catastrophic failures, and they are generated by fracture mechanics because of an uneven distribution of forces in objects that already contain cracks or defects. In addition, the application of a load on any solid initially results in a reversible elastic deformation that is followed by a fracture with little or no plastic deformation or a fracture preceded by considerable plastic deformation. A fracture is defined as the separation or fragmentation of a solid body into two or more parts under the action of loads [15]. Fracture toughness is the resistance of the ceramic material to crack growth, which is primarily controlled by the mechanism of transformation resistance, depending on grain size, sintering temperature, and yttrium content. Any ceramic material can fail from a fracture associated with the brittleness of the material that originates during processing or from its use. Because of the presence in its structure of a mechanism called “transformation resistance”, the mechanical properties of zirconium have been improved [16]. This mechanism plays an important role in fracture toughness, having the purpose of avoiding the formation and propagation of cracks [17].

The sintering process is a heat treatment to which zirconium dioxide is exposed. It is a fundamental treatment since in addition to influencing the final geometric shape, it improves the mechanical and optical properties. Therefore, companies have developed varieties of furnaces with protocols adjusted to different thermal units; such is the case of InFire HTC Speed, which with a maximum sintering temperature of 1650 °C can take up to 8 h of slow sintering [18], and CEREC SpeedFire, which, depending on the size and volume of the zirconia restoration, can take 15 min, reaching a fast sintering temperature of 1600 °C [19].

Therefore, different protocols have been proposed to shorten clinical and laboratory times, pursuing the philosophy of CAD/CAM technology with chairside systems, with heat treatment being a very important variable to consider regarding its influence on mechanical properties [20,21,22,23].

The purpose of this study was to compare the fracture strength of monolithic zirconium dioxide subjected to a sintering process in two different furnaces, following the manufacturer’s specifications in relation to the applied temperature and sintering time. With this objective, the aim was to demonstrate whether the sintering units influence the mechanical properties of the materials. The null hypothesis of this study was that no statistically significant differences would be found in the fracture strength values of translucent monolithic zirconium dioxide subjected to different sintering units.

## 2. Materials and Methods

In this in vitro study, 40 CAD/CAM restorations fabricated with pre-sintered translucent monolithic zirconia blocks (InCoris TZI, Dentsply-Sirona, Bensheim, Germany) were analyzed. The 40 crowns were randomly assigned to two groups of 20 samples each: Group 1 (SpeedFire, Dentsply-Sirona, Bensheim, Germany) and Group 2 (InFire HTC Furnace, Dentsply-Sirona, Germany).

### 2.1. Sample Preparation

**Figure 1:**
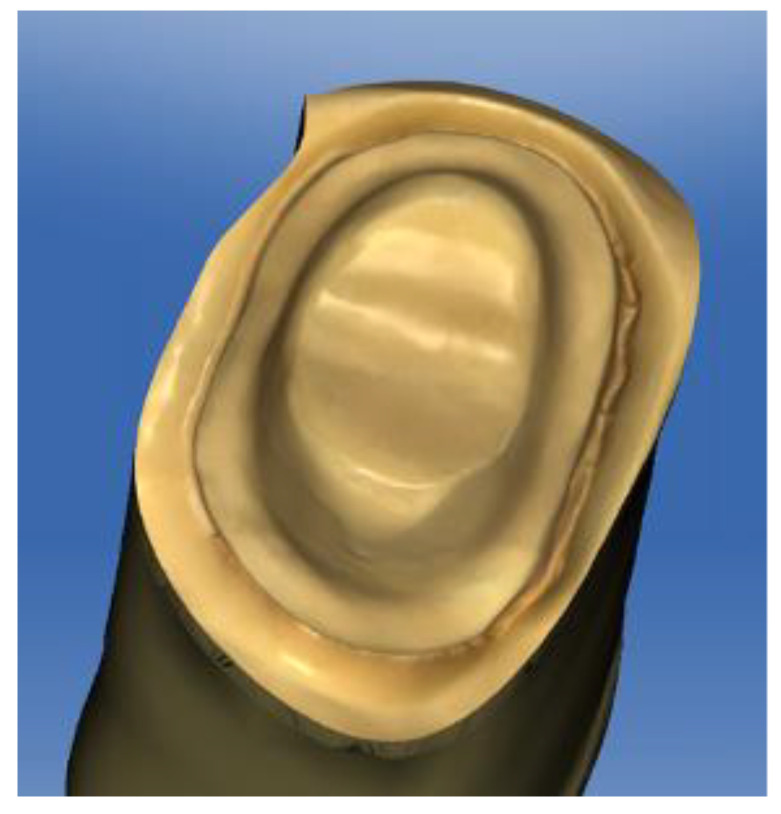
Preparation of the abutment.

A full-crown preparation of the model of a right upper first premolar (Figure 1) with a 1 mm termination line with a rounded shoulder was used. The tooth preparation was scanned using a digital intraoral scanner (PrimeScan, Dentsply-Sirona, Bensheim, Germany). The restoration was designed using CAD software (CEREC 5.0, Dentsply-Sirona, Bensheim, Germany), and the restoration was designed with standardized parameters and dry-machined on a compact milling machine (MCXL Premium, Dentsply-Sirona, Bensheim, Germany).

### 2.2. Thermocycling

All samples were subjected to a thermocycling process; the cycles used were 5000 cycles. Thermal cycling with extreme temperatures of 5 and 55 °C in distilled water (residence time: 25 s, pause time: 10 s) was performed in the computerized thermocycling unit (Thermocycler™, SD Mechatronik, Feldkirchen-Westerham, Germany). The samples were placed in a thermal cycler container and then dried and inspected for cracks, chips, or fractures after each loading phase. No damage in the specimens was found after thermocycling.

### 2.3. Sintering

The sintering of the zirconium dioxide restorations was carried out in the furnace determined according to the assigned group. The restorations of Group 1 were sintered in a fast sintering furnace (CEREC SpeedFire, Dentsply-Sirona, Bensheim, Germany) whose duration depends on the software that determines the time and maximum temperature automatically according to each restoration, which in this case, was 18 min at a maximum temperature of 1600 °C. As all of the crowns were identical and standardized, the furnace always showed the same time. The restorations of Group 2 were sintered in a slow sintering furnace (InFire HTC, CEREC SpeedFire, Dentsply-Sirona, Bensheim, Germany) with a sintering time of 8 h at a maximum temperature of 1650 °C in a program preset for the material.

### 2.4. Fracture Test

**Figure 2:**
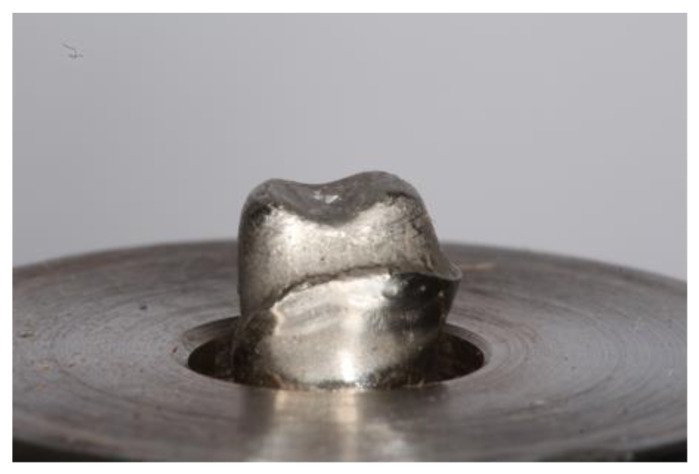
Abutment obtained in cobalt–chromium.

**Figure 3:**
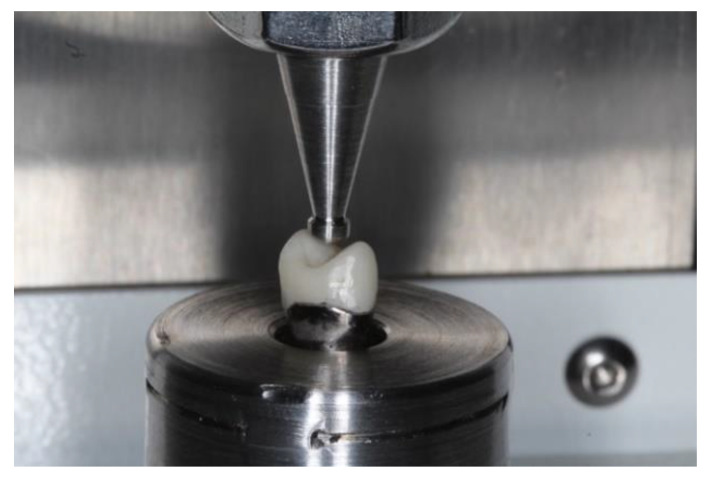
Loading of the specimen seated on the cobalt chromium alloy abutment using a universal testing machine.

To determine the fracture strength of the material after the sintering process, a universal compression machine (SuperL 120, Tinius Olsen, Horsham, PA, USA) was used. A replica of the abutment was obtained in a cobalt–chromium alloy to seat each specimen for testing purposes (Figure 2). The test was performed with a maximum load cell recorded in Newtons (N) and a displacement control with a speed of 1 mm/min. Fracture of each specimen was performed using a standard tip (diameter = 2 mm) placed on the occlusal surface of each specimen. During the compression test, the load and displacement experienced by each specimen at the moment the tip touched the material and caused the fracture were continuously recorded. Each group of specimens was given a specific identification number (Figure 3). The maximun load force until the failure was registered by the software (Horizon, Tinius Olsen, Horsham, PA, USA) connected to the universal machine.

### 2.5. Data Processing

The data for each group were compiled in an EXCEL™ sheet (Microsoft, Redmond, WA, USA). They were then entered into a database in the SPSS 22 program (Statistical Package for Social Science, IBM Corporation, Armonk, NY, USA). For the descriptive statistical analysis, the mean standard deviation and standard error mean were calculated. Fracture resistance results were normally distributed. Student’s *t* test was used to compare the mean fracture strength between Groups 1 and 2.

### 2.6. Weibull’s Modulus

In the present study, once the resistance values were obtained in megapascals (MPa), they were sorted in ascending order, and an analysis protocol based on the Excel program was used, by means of the “Microsoft Excel for Weibull Analysis” guide. The results of the Weibull modulus were expressed as the end of the slope in a single value.

## 3. Results

**Figure 4:**
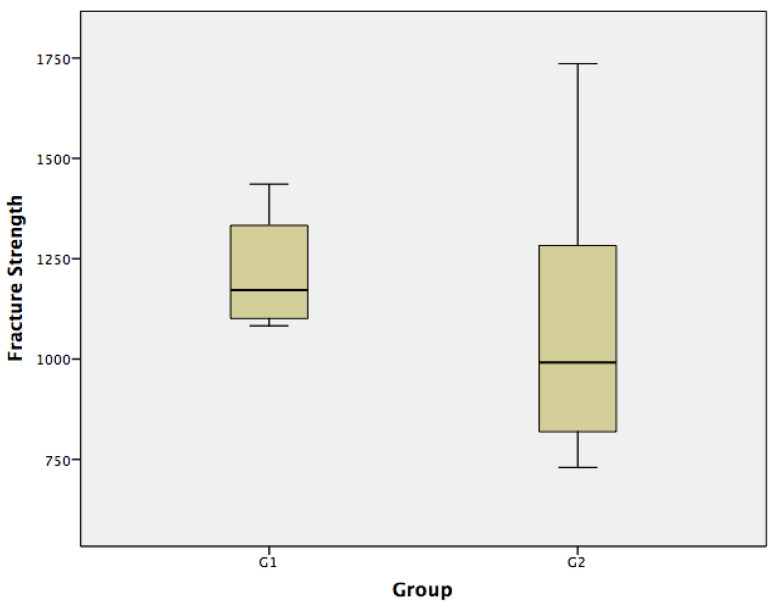
Box plot for the data of the groups studied.

Descriptive statistics showed the load values as seen in Table 2 for Group 1 $[\bar{x}=$ 1222.80, σ 136.91 N] and Group 2 [$\bar{x}=$1068.50, σ 316.39 N] (Figure 4). In the normality test, both Group 1 and Group 2 presented a normal distribution (>0.05).

According to Student’s *t* test, the hypothesis that there is no significant difference between the fracture resistance means of Group 1 and Group 2 was not supported. Resistance to fracture of Group 1 and Group 2 (*p*-value: 0.182) was accepted. Therefore, based on the results obtained, Group 1 and Group 2, according to their compression loads, did not present statistically significant differences.

**Figure 5:**
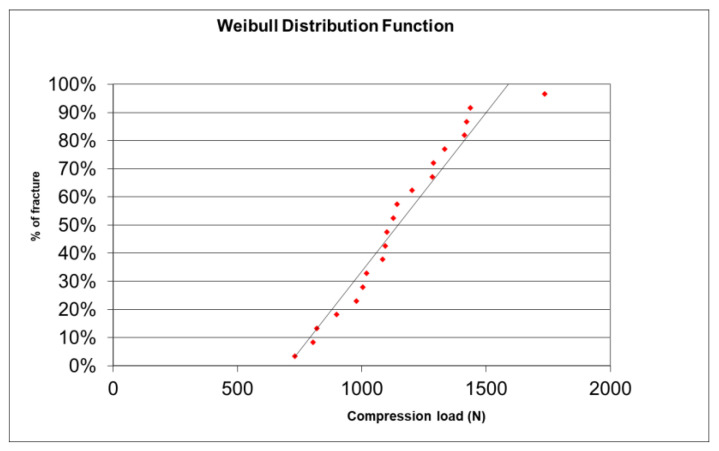
Weibull’s distribution function.

**Figure 6:**
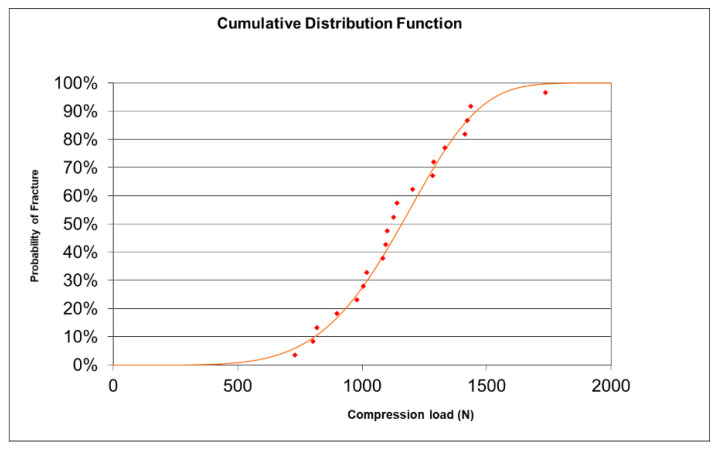
The cumulative distribution function (CDF) on which fracture probabilities can be calculated is shown. For example, the probability of fracture at 1000 N is 27.5% or 93.1% at 1500 N.

Regarding the Weibull modulus, the slope of the straight line that intercepts most of the points on the Weibull Plot is also the Shape Factor β (5.227) and indicates what type of probability distribution it approximates (normal, lognormal, exponential, etc.) (Figure 5). The characteristic reliability, η, is the time expected to be 63.2% of the Mean Rank of the straight line and corresponded to a load of 1242 N (t = η). A cumulative distribution function was determined (Figure 6). This 63.2% was true for all Weibull distributions, regardless of the shape parameter β.

**Figure 7:**
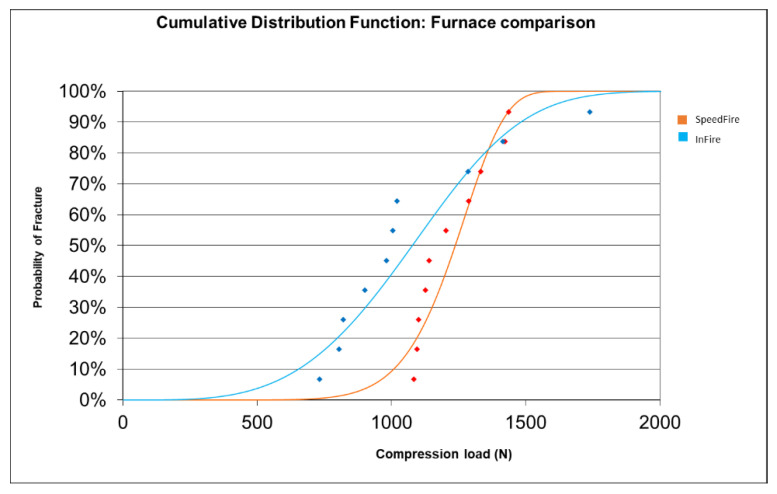
Comparative analysis of the CDF between the groups studied.

The results comparing the CDF for the two furnaces are shown in Figure 7.

The CDF for the SpeedFire and InFire furnaces were also compared: it was observed that Furnace 1 (Spedd Fire) was more resistant to load until fracture than Furnace 2 (InFire) up to 1365 N (orange curve below blue, lower probability of fracture for F1). At 1365 N and above, it was Furnace 2 (InFire) that resisted more load to fracture (blue curve below orange, lower fracture probability for F2).

## 4. Discussion

In the present study, no statistically significant differences were found between the groups studied. Therefore, it can be deduced that mechanical properties such as fracture strength are not affected by the sintering time to which they are subjected. This property is a weak point of dental ceramics; although they have been widely used for their biocompatibility and aesthetic potential, owing to their innate fragility, they present this limitation in their clinical application. Therefore, materials such as zirconium dioxide with high toughness (6–10 MPa/m^1/2^), because of their fully crystalline microstructure and thanks to the presence of a resistant transformation mechanism, present higher fracture strength values in relation to other ceramic materials by preventing fracture propagation [13,24]. One study, for example, reported that the predominant failure between monolithic zirconia and layered lithium disilicate crowns was the veneering ceramic, and that the highest fracture strength was presented in the monolithic zirconia crown [5]. Previous studies reported that the highest chipping rate was in crowns with zirconia frameworks veneered with feldspathic or translucent glass–ceramic materials using the manual layering technique [25,26,27]. In another study, the usefulness of CAM technology for milling ceramic blocks compared to pressing lithium disilicate ceramics on zirconia frameworks in fixed prostheses was reported, observing comparable fracture strength to monolithic zirconia fixed prostheses and higher fracture strength than monolithic lithium disilicate [28].

In this study, ceramic crowns were machined with monolithic pre-sintered zirconia blocks using a CAD/CAM system, producing the same design in each specimen. With monolithic crowns, the benefit of CAD/CAM technology was obtained compared to the manual veneering technique by using a high quality material with a minimum of defects, such as bubbles, difficult repeatability, and systematic reproduction of details [1]. The CAD/CAM system allows the production of standardized restorations with reduced production costs, labor, and time, where the chairside system is a more integrated process with faster results. Kallala et al. [29] concluded in their study that CAD/CAM systems are a promising technology that, in addition to saving time, are less dependent on complicated laboratory processes and enable better communication with the patient. The tooth preparation, the crown design, and the method of fabrication influence the strength load offered by the ceramic material. These design variables cannot be evaluated independently because an alteration in any one of them can cause changes in the other variables [30]. A previous study, for example, reported significant differences in the design of milled prosthetic restorations with CAD/CAM systems [31]; therefore, in addition to achieving an adequate marginal adaptation within the digital flow with digital scans, the risk of errors from the impression material, which undergoes deformations during polymerization, can be avoided. Conventional methods used to sinter monolithic zirconia include high-temperature processes and long heating times that consume energy and time [9]. The most common sintering temperature range is considered to be from 1400 to 1600 °C, depending on the manufacturer [32,33]. Sintering parameters can also affect the microstructure and material properties [6]. The sintering temperature of 3Y-TZP zirconia is typically between 1350 and 1550 °C [33,34,35]. The zirconium dioxide used in this study reached a sintering temperature of 1650 °C in the InFire HTC furnace and a maximum of 1600 °C for the CEREC SpeedFire furnace, so the temperatures were within the parameters. In general, a fine and uniform grain structure with high density is required for the material to achieve adequate mechanical properties. Inokoshi et al. [34] addressed microstructural analysis and reported significant grain growth in 3Y-TZP zirconia-based ceramics at high temperatures (1650 °C) with increasing exposure time (2–4 h). It should be emphasized that the grain size of 3Y-TZP zirconia is even more sensitive in the martensitic transformation of the tetragonal and monoclinic phases, equally influencing its mechanical properties. Borrel et al. [35] showed that the mechanism of grain growth in 3Y-TZP zirconia during microwave sintering differed from that obtained by conventional sintering without pressure. Therefore, it is important to establish that furnaces without a vacuum mechanism can affect the final results of the material. Both temperature and sintering time affect the grain size of the material; therefore, if the sintering temperature is high and the sintering time longer, the grain size will be larger [36,37]. Several studies have provided evidence that zirconia grain size reduction is possible, more effectively, through sintering in less than 4 h [33,37]. Currently, there are several furnaces that allow restorations to be carried out in the same day. Another study reported that dental zirconia ceramics can be rapidly sintered at 1580 °C with a dwell time of less than 20 min to achieve a clinically adaptable performance that is feasible for chairside restorations in a single visit [38]. By using a fast sintering furnace in this study, the requirements of the chairside clinical technique were achieved without affecting the mechanical properties of the ceramic. In addition, this method offered several advantages over the conventional method, including improved productivity, time savings, and lower energy consumption.

Fracture strength is considered to be one of the most important factors for the long-term success of dental restorations. Ceramic restorations are subjected to chewing forces and other factors in the oral cavity that can affect the mechanical properties and their strength [39]. It has been reported that occlusal strength depends on gender, age, and strategic position in the arch (anterior or posterior), with a considerable difference in values. However, a dental restoration should withstand an occlusal force greater than 1000 N because the occlusal force exceeds this value in parafunction [24]. According to the result of the load test in this study (Table 2), the two groups showed values that exceeded the range of chewing force reported in the literature. The findings of this study reinforced what has been previously reported in load tests of both anterior and posterior teeth, although the results were highly variable because each of them presented different experimental methods [37,38]. Among other factors affecting strength, the modulus of elasticity of the material used for the die is an important factor [40,41]. One study reported fracture strength exceeding 2000 N in zirconia crowns of upper central incisors cemented to a metal die with resin cement [42]. In another study, a load test was applied to 20 monolithic zirconia crowns of premolar teeth cemented to models with epoxy resin, where the crowns presented a load resistance of 3200 N [33]. Other authors have evaluated the fracture strength of ceramic crowns on different support materials, considering that increasing the modulus of elasticity of the material will increase the fracture strength of posterior all-ceramic crowns [37]. This agrees with the observation of Scherrer and de Rijk [42], who reported in their study that the fracture loads of all-ceramic crowns increased with the elastic modulus of the supporting structure.

In ceramic materials there is a considerable dispersion of defects, i.e., different sizes. This means that parts made from the same ceramic material may fail at different values of the maximum applied force. The Weibull modulus determines the arrangement of material defects based on the material based on the weakest link theory, in which it is assumed that a given volume of ceramic under a uniform load would fail the most in the area with the greatest imperfection, allowing evaluation of its reliability. The table showing the cumulative distribution function (CDF) shows the fracture probabilities. For example, the probability of fracture at 1000 N is 27.5% or 93.1% at 1500 N. According to our results, it was observed that Furnace 1 (Spedd Fire) was more resistant to load until fracture than Furnace 2 (InFire) up to 1365 N (orange curve below blue, lower probability of fracture for F1). At 1365 N and above, it was Furnace 2 (InFire) that withstood more load to fracture (blue curve below orange, lower fracture probability for F2).

On the other hand, although a natural tooth could have replicated the clinical environment more accurately if it had been chosen as the abutment, in this study a metal alloy abutment was considered since natural teeth have different sizes, shapes, and qualities, and thus the preparation material is difficult to standardize. It was also considered that natural teeth with a lower elastic modulus may fracture near the cervical area. Therefore, in the present study, a cobalt–chromium alloy abutment with higher elastic modulus and fracture strength was used to test the fracture strength consistent with material used for the abutment in another study [43]. Among other parameters considered, in a previous study, fracture strength was evaluated in relation to crown thickness and the luting agent [41]. Accordingly, 0.5-mm-thick monolithic zirconia crowns provided sufficient strength, regardless of the type of cementation. In contrast, the fracture strength of 0.2-mm-thick cemented zirconia crowns was low for clinical application, although adhesive bonding improved performance and stability. According to the information obtained, the fracture strength test values after cementation were significantly higher compared to our study using groups with uncemented cores, so the material and cementation technique did not influence the mechanical behavior of the final ceramic restorations [11]. The significant increase in fracture load data for cemented ceramic cores may be related to both the higher mechanical properties of the resin-luting material and the bond established at the interfaces between the ceramic, luting material, and abutment [40,41]. Different tests have been used to analyze the mechanical behavior of dental materials, including static load and fatigue tests. Laboratory tests apply static loads until failure of the material using a universal machine, representing its behavior on a force–displacement curve and recording the maximum applied load. These tests provide information on the strength of the material, the potential risk of failure, and the deformation of the material. However, they cannot sufficiently predict the long-term performance of dental restorations. Badawy et al. [44] mentioned in their study the importance of knowing the fracture toughness of dental ceramics, which by nature are brittle and have a higher susceptibility to fracture under stress. A restorative material with high fracture toughness shows improved fracture strength and longevity. Zirconia ceramics are known for their higher load resistance and toughness compared to other ceramics. Clinically, mechanical failure of dental prostheses can occur long after installation. Damage accumulates from repetitive contact between maxillary and mandibular teeth with the result that the survival rate of the prosthesis is reduced [45]. Therefore, future studies should reproduce experimental conditions similar to clinical situations to create the failure pattern in real clinical practice. This study has some limitations, such as in vitro study and not including more materials from other commercial companies to evaluate the effects of different sintering times. The time taken to produce a restoration in the clinic and laboratory is of utmost importance. Therefore, in this study, it has a high impact to know that by using a furnace with shorter sintering times, the mechanical properties of the material are not altered. Other factors to consider are the area of chewing and the size of the prosthesis. For example, a single tooth in the posterior sector can be quickly synthesized. In fact, its strength will be greater if it is made in a fast sintering furnace. However, a restoration that requires greater strength, such as a fixed dental prosthesis, should undergo slow sintering to improve its reliability.

Within the limitations of this study, only the fracture resistance was tested. It was not our objective to structurally characterize the material, since there is sufficient information in this regard, so a fractographic analysis was not performed. Some studies of this type have not performed this analysis either [46,47]. Finally, more studies that can perform a structural analysis are needed to better understand what happens to the materials after applying various heat treatments to them.

## 5. Conclusions

Fractured crowns with loads higher than those considered clinically relevant suggest that monolithic zirconia crowns can withstand even parafunctional masticatory forces. Since the oral environment is much more complex and there are different influential factors, it is difficult to use mechanical load test results alone. It is important to adopt a sintering temperature appropriate to the size of the prosthesis together with an adequate clinical time, so that the properties of the ceramic material are not affected. Although no significant difference was found in the present study between the two groups in which the mechanical strength of the material was not compromised, it can be concluded that the rapidly sintered material has a survival limit. It is further suggested to evaluate the long-term clinical performance of restorations obtained with different sintering times.

## Figures and Tables

**Table 1:** Composition and characteristics of zirconia [10].

Zr Generation	Composition	Characteristic	Sintering Profile
First	3Y-TZP (3 mol % partially stabilized zirconia), Al_2_O_3_ (≈0.25%)	High opacity Grain size: ~0.5–1 μm	Modification of crystalline phases with sintering time
Second	3Y-TZP (3 mol % partially stabilized zirconia), Al_2_O_3_ (≈0.05%)	Poor aesthetics Grain size: ~0.5–1 μm	High temperature
Third	5Y-PSZ (5 mol % partially stabilized zirconia)	Improved translucency Reduced strength and toughness Grain size: ~1.5 μm	High temperature
Fourth	4Y-PSZ (4 mol % partially stabilized zirconia)		

**Table 2:** Descriptive statistical results for the two groups.

Samples	Group	Load (N) Mean	Standard Deviation	Average Standard Error
20	1: Speed fire	1222.80	136.90	43.29
20	2: InFire HTC	1068.50	316.38	100.05
